# Comparison of Biochemical Recurrence After Robot-assisted Laparoscopic Radical Prostatectomy with Volatile and Total Intravenous Anesthesia

**DOI:** 10.7150/ijms.40958

**Published:** 2020-02-04

**Authors:** Na Young Kim, Won Sik Jang, Young Deuk Choi, Jung Hwa Hong, Sewon Noh, Young-Chul Yoo

**Affiliations:** 1 Department of Anesthesiology and Pain Medicine, Anesthesia and Pain Research Institute, Yonsei University College of Medicine, Seoul, Republic of Korea.; 2 Department of Urology and Urological Science Institute, Yonsei University College of Medicine, Seoul, Republic of Korea.; 3 Department of Policy Research Affairs National Health Insurance Service Ilsan Hospital, Goyang, Gyeonggi-do, Republic of Korea.

**Keywords:** Prostate cancer, recurrence, propofol, volatile, anesthesia.

## Abstract

**Aims**: Recurrence after cancer surgery is a major concern in patients with cancer. Growing evidence from preclinical studies has revealed that various anesthetics can influence the immune system in different ways. The current study compared the long-term biochemical recurrence of prostate cancer after robot-assisted laparoscopic radical prostatectomy (RALP) in terms of selection of anesthetic agent between total intravenous anesthesia (TIVA) with propofol/remifentanil and volatile anesthetics (VA) with sevoflurane or desflurane/remifentanil.

**Methods**: We followed up oncologic outcomes of patients who underwent RALP from two previous prospective randomized controlled trials, and the outcomes of those who received TIVA (n = 64) were compared with those who received VA (n = 64). The follow-up period lasted from November 2010 to March 2019.

**Results**: Both TIVA and VA groups showed identical biochemical recurrence-free survivals at all-time points after RALP. The following predictive factors of prostate cancer recurrence were determined by Cox regression: colloid input [hazard ratio (HR)=1.002, 95% confidence interval (CI): 1.000-1.003; P = 0.011], initial prostate-specific antigen level (HR=1.025, 95% CI: 1.007-1.044; P = 0.006), and pathological tumor stage 3b (HR=4.217, 95% CI:1.207-14.735; P = 0.024), but not the anesthetic agent.

**Conclusions**: Our findings demonstrate that both TIVA with propofol/remifentanil and VA with sevoflurane or desflurane/remifentanil have comparable effects on oncologic outcomes in patients undergoing RALP.

## Introduction

A major concern among patients with cancer is recurrence after cancer surgery. Aside from several well-documented factors that synergistically influence the risk of long-term cancer recurrence, recent studies have shown that anesthetic agents might affect the postoperative prognosis of cancer due to its unfavorable effect on the immune system 1, 2, and has led to a renewed interest in this field 3.

Propofol has been reported to exhibit positive immunomodulatory effects 3-5, and better survival has been reported after cancer surgery with propofol-based total intravenous anesthesia (TIVA) compared to volatile anesthesia (VA) 6-9. However, other recent studies have yielded different results regarding the influence of anesthetic agents on the recurrence of breast cancer 10, 11, and different oncologic outcomes have been demonstrated depending on the type of cancer 7, 11-14.

Currently, the most common malignancy among males in the United States is prostate cancer 15, with over 160,000 new cases diagnosed in 2018 and almost 30,000 resultant deaths 16. Until now, there have been no studies of the influence of anesthetic agents on the recurrence of prostate cancer. We previously published two prospective randomized controlled trials, which were conducted to compare postoperative nausea and vomiting (PONV) and changes in intraocular pressure (IOP) in patients who underwent robot-assisted laparoscopic radical prostatectomy (RALP) under general anesthesia with either propofol-based TIVA or sevoflurane-based VA 17, 18. The patients who participated in these two studies underwent RALP 7-9 years ago, providing a unique opportunity to assess their cancer status and long-term survival.

Thus, this study aimed to compare the effects of VA with sevoflurane or desflurane/remifentanil and TIVA with propofol/remifentanil on long-term oncologic outcomes, such as biochemical recurrence (BCR), in patients with prostate cancer after RALP.

## Materials and Methods

### Study design and participants

Detailed descriptions of the design of the previous trials (PONV and IOP) have been published previously 17, 18. PONV and IOP were randomized controlled trials that evaluated and compared the effects of VA with sevoflurane or desflurane/remifentanil and TIVA with propofol/remifentanil on PONV and the changes of IOP in patients undergoing RALP. Sixty-two patients between November 2010 and May 2011, and 66 patients between May 2011 and Mar 2012, were enrolled in the PONV and IOP trials, respectively. Additional ethics approvals for the follow-up study were obtained from the institutional review board (IRB) and hospital research ethics committee (Yonsei University Health System, Seoul, Korea; IRB protocol No. 4-2019-0313, approved on 23 May, 2019). The need for informed consent from the patients was waived. Patients were followed up from November 2010 to March 2019. Additional criterion for enrolment in the follow-up study included patients who underwent complete surgical excision of prostate cancer without any surgical complications. Patients with uncertain clinical conditions or incomplete follow-up data were excluded. For this follow-up study, BCR status, prostate specific antigen (PSA) levels, and TNM staging of tumor were collected at time points after the original trials.

### Anesthesia protocol

Premedication was performed with 0.05 mg/kg midazolam and 0.2 mg glycopyrrolate 1 hour before and immediately before the initiation of anesthesia, respectively. Standard monitoring with non-invasive blood pressure, electrocardiography, and saturation was conducted. Anesthesia was induced with propofol and remifentanil in both groups. After loss of consciousness, 0.6 mg/kg rocuronium was administered to facilitate tracheal intubation. Controlled ventilation was performed with 40% oxygen in air to maintain end tidal carbon dioxide (CO_2_) at 35-40 mmHg during the surgery, and a positive end-expiratory pressure of 5 cm H_2_O was applied. Body temperature was maintained at 36-37°C using a forced-air warming system. For continuous blood pressure monitoring, 20-G radial artery catheterization was performed in all patients.

In the VA group, anesthesia was induced with an intravenous bolus of propofol (1.5 mg/kg) and maintained with sevoflurane (1.5-2.5%) or desflurane (4-7%). In the TIVA group, propofol was administered using a TCI pump (Orchestra® Base Primea, Fresenius Vial, France) according to the Marsh model. 19 The target plasma concentration of propofol was maintained within 2-5 μg/mL. Remifentanil was administered to all patients using a TCI pump, according to the Minto model 20, and the effect-site concentration was adjusted to within 2-5 ng/mL. The anesthetics administered to each group were titrated to maintain mean arterial pressure (MAP) and heart rate within 20% of baseline values and to provide adequate depth of anesthesia. A bispectral index score (BIS) monitor (Aspect A-2000®, Aspect Medical System Inc., Newton, MA, USA) was used so that the BIS could be maintained within the range of 40-60. Additional boluses of 0.15 mg/kg rocuronium were administered as needed to maintain adequate intraoperative neuromuscular blockade. During the operation, CO_2_ pneumoperitoneum was induced to maintain a mean (standard deviation, SD) intra-abdominal pressure of 15 (5) mmHg using a CO_2_ insufflator in a 30° Trendelenburg position. The Trendelenburg angle was accurately adjusted by the operating table controller, which showed the tilted angle in numbers.

All patients received 0.3 mg ramosetron intravenously 20 minutes before the end of surgery, and postoperative pain was controlled using intravenous patient-controlled analgesia (PCA), consisting of 20 μg/kg fentanyl mixed with normal saline to a total volume of 100 ml, administered at a basal rate of 2 ml/h, with a bolus dose of 0.5 ml and a 15-min lockout time. At the end of surgery, reversal of neuromuscular blockade was performed with 50 μg/kg neostigmine and 10 μg/kg glycopyrrolate intravenously. After extubation and when fully awake, the patients were transported to the post-anesthesia care unit.

### Statistical Analysis

The primary endpoint of the study was the effects of VA with sevoflurane or desflurane/remifentanil and TIVA with propofol/remifentanil on BCR in patients after RALP. Patients were followed up with serum PSA tests at 3-month intervals for the first 2 years, at 6-month intervals for the next 3 years, and annually thereafter. BCR was defined as two consecutive rises in serum PSA levels ≥0.2 ng/ml at any time postoperatively. Continuous variables are presented as means (SD), and categorical variables are presented as numbers of patients (percentages). Continuous variables were compared using independent *t*-tests, and categorical variables were compared using the chi-square test or Fisher's exact test. Kaplan-Meier curves were calculated based on the occurrence of BCR for each period (12, 24, 36 months, and overall), and the groups were compared using the log-rank test. Cox proportional hazard regression was performed to identify associated independent factors, and the results are presented as hazard ratios (HRs) and 95% confidence intervals (CIs). SAS version 9.4 (SAS Institute Inc., Cary, NC, USA) was used for all statistical analyses. A *p*-value < 0.05 was considered statistically significant.

## Results

**Figure 1:**
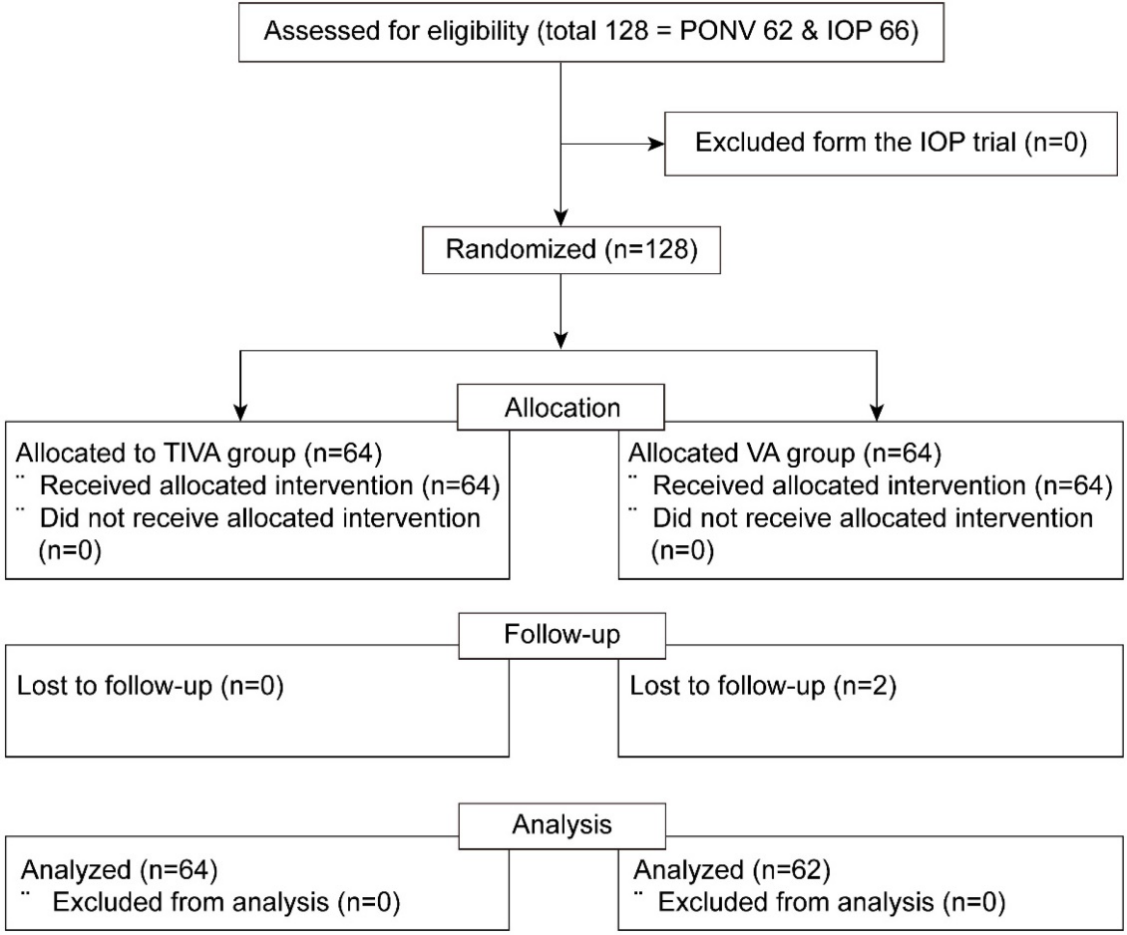
Consort flow diagram. PONV = postoperative nausea and vomiting; IOP = intraocular pressure; TIVA = total intravenous anesthesia; VA = volatile anesthesia.

Out of 128 patients who were enrolled in both prospective randomized trials (62 and 66 eligible patients in the PONV and IOP trials, respectively), two patients with follow-up loss were excluded from the VA group. The final sample for analysis included 126 patients who had been assigned to either the TIVA (n = 64) or the VA (n = 62) group (Figure 1).

Table 1 shows the distribution of demographics and perioperative variables in both groups. As is expected from the randomized study design, baseline characteristics of the two groups were comparable. In the VA group, two volatile agents were used: 31 patients received sevoflurane and 31 patients received desflurane, with BCR observed in 13 patients (42%) receiving sevoflurane and in 15 patients (48%) receiving desflurane. There was no significant difference in the incidence of BCR between the two volatile agents in the VA group (P = 0.799).

The incidences of BCR and pathological variables are presented in Table 2. The number of patients who presented BCR was 28 (45.2%) in the VA group and 25 (39.1%) in the TIVA group, with no significant difference between the two groups. Similarly, the number of patients with positive radiographic progression did not differ between the two groups. Initial PSA levels, Gleason score, surgical margin status, pathological tumor stage, and lymph node metastasis were also similar in both groups.

**Figure 2:**
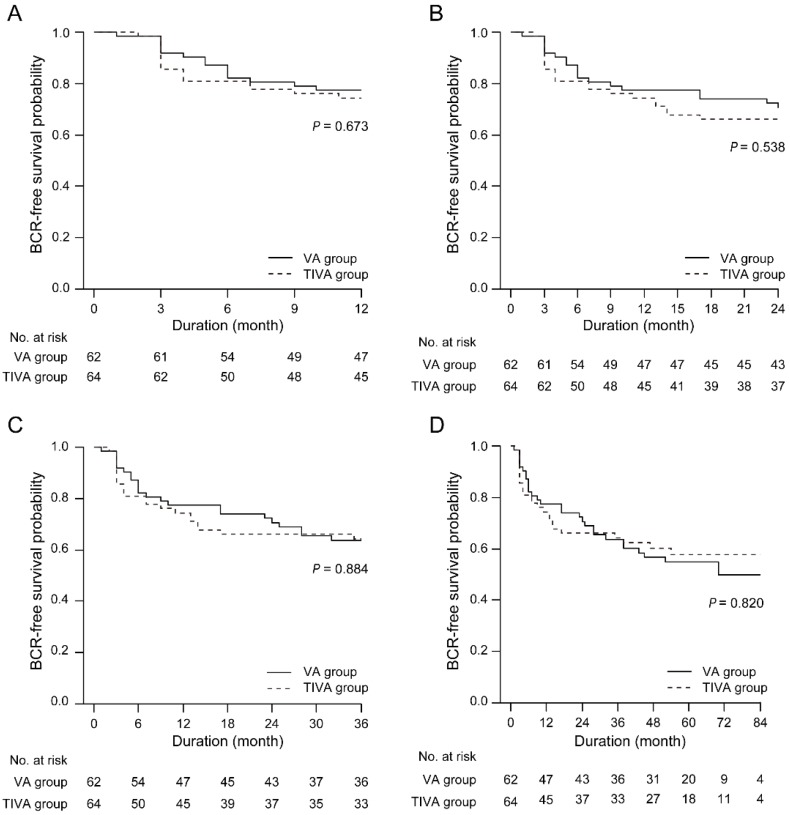
BCR-free survival between the TIVA and VA groups at any follow-up point after RALP. IOP = intraocular pressure; TIVA = total intravenous anesthesia; VA = volatile anesthesia; BCR = biochemical recurrence.

BCR-free survival was identical between the VA and TIVA groups at any follow-up point after RALP (Figure 2).

Cox regression identified significant predictors of prostate cancer recurrence after RALP as colloid input (HR=1.002, 95% CI:1.000-1.003; P = 0.011), initial PSA level (HR=1.025, 95% CI:1.007-1.044; P = 0.006), and pathological tumor stage 3b (HR=4.217, 95% CI:1.207-14.735; P = 0.024), but there was no dependence found on different general anesthetics (Table 3).

## Discussion

This is the first study to compare the effects of VA with sevoflurane or desflurane/remifentanil and TIVA with propofol/remifentanil on oncologic outcomes in patients after RALP. An additional strength of this study is that we analyzed 7-9 years of outcomes in patients who were randomly assigned to either the VA or TIVA groups.

It has been well documented that metabolic and neuroendocrine changes due to perioperative stress cause significant depression of cell-mediated immunity. Inevitably, this can bring out micro-seed tumor cells during surgery which can avoid host immune surveillance, eventually resulting in tumor recurrence 8. In addition, there is growing evidence from preclinical studies to show that volatile anesthetic agents may alter immune processes through the attenuation of NK cell cytotoxicity 21, cytokine release, and chemotaxis of immune cells 22, 23. Moreover, several studies have shown that volatile anesthetic agents can have a direct effect on tumor cells and induce mitogenesis, angiogenesis, and metastasis of tumors, which is associated with increased expression of insulin-like growth factor, vascular endothelial growth factor, and hypoxia-inducible factor (HIF) protein 24-27.

In contrast, propofol appears to increase the activity of cytotoxic T-lymphocytes 28 and not suppress NK cell activity *in vitro* 3. In addition, propofol directly prevents HIF activation 29, and may exert anti-tumor effects mediated through inhibition of the cyclooxygenase pathway 30. It has also been reported that propofol has an inhibitory effect on the sympathetic nervous system, which attenuates catecholamine release 31. The nervous system is a primary actor in early and late cancer development. Many cancers (e.g., colon and prostate) present a specific growth pattern called “perineural invasion” which is driven by neurons 32. The growth of some cancers, such as prostate cancer, pancreatic cancer, and breast cancer, is associated with “neurogenesis” and abundant infiltration of the tumor by the autonomic nervous system 33. The prostate has a sympathetic nerve supply that is 5 or 6 times greater than that of the other chromaffin organs, making it particularly susceptible to these effects 34. The stress response results from stimulation of the sympathetic nervous system, which enhances tumor growth through immunosuppression and beta-adrenergic stimulation of tumor cells 33. In animal studies, sympathectomy has been reported to slow the progression of cancer, and human retrospective data have indicated lower rates of recurrence and mortality among patients with cancer taking beta-blockers 35. Thus, we envisaged favorable long-term oncologic outcomes in the TIVA group owing to the comparatively safer effects of propofol, such as the relative preservation of cellular immunity and inhibition of sympathetic activity. However, our results showed no difference in the rate of recurrence after RALP between the VA and TIVA groups. In addition, there was no difference between these groups after subgroup analysis of volatile anesthetics.

There have been many clinical studies on this topic, but these were retrospective studies. Previous studies in patients with colorectal and esophageal cancer showed better prognosis in patients receiving TIVA with propofol 10-12. However, in studies of lung and breast cancer surgery, there were no differences in oncologic outcomes, which is consistent with the current results 8,9,14,36. It has been shown that expression of catecholamine associated with stress, such as norepinephrine, promotes cell migration in colon, prostate, ovarian, and breast cancer cells 37. Similarly, according to the type of cancer, volatile anesthetics or propofol may have markedly different influences on oncologic outcomes. To date, however, no studies have been conducted to compare the effects of volatile anesthetics or propofol on oncological outcomes according to the various cancer cell types. Therefore, more studies are required in this area.

While the impact of opioid use on recurrence is debatable, opioids such as fentanyl and morphine have been reported to promote poor oncologic outcomes in terms of immunity and angiogenesis 38, 39. Regarding the unfavorable tumor microenvironment caused by opioids, intraoperative infusion of remifentanil may have affected the recurrence rate after RALP in our study. Although a previous study provided evidence that a low dose infusion of remifentanil does not affect cellular immunity status 40, this study targeted only healthy volunteers and did not include patients with reduced immunity or immunocompromised patients, such as patients with cancer. The dose was also lower than that typically administered during surgery. In animal models of breast cancer, early administration of morphine did not affect the growth of cancer cells, but morphine administered after cancer progression reduced the survival rate, as morphine directly acts on µ-opioid receptors (MOR) expressed in the tumor, thereby promoting the growth of cancer cells 41. In recent studies, it has been shown that the increased expression of MOR in patients with cancer is associated with worse prognosis. A recent mediation study showed that twice as much MOR is expressed in the tissues of patients with metastatic lung cancer compared to patients with lung cancer 42. In addition, a study of patients with advanced prostate cancer indicated a significantly shorter progression-free survival in patients with higher MOR expression in cancer tissues and greater morphine requirements after cancer diagnosis 43. Considering the impact of opioids on cancer growth, the continuous infusion of remifentanil in both groups could have been another confounding factor for the negative outcomes in the current study.

Finally, in previous reports that showed favorable effects of propofol on oncologic outcomes, most cases were combined with epidural analgesia, except one that involved patients undergoing gastrectomy 8, 9, 13, 14, 36. However, in recent studies which did not apply regional or neuraxial analgesia, there was no benefit of propofol-based TIVA for long-term oncologic outcomes after non-small cell lung cancer and breast cancer surgery 10-12. The majority of studies which showed better oncologic outcomes associated with propofol-based TIVA applied regional or neuraxial analgesia for pain control, and consequently, small dose of opioids were used during surgery. Opioids may therefore be a significant confounding factor for confirming the influence of propofol on oncologic outcomes. Thus, there are many difficulties in obtaining accurate results, and well-controlled prospective clinical studies of these confounding variables are required.

There are several limitations to the current study. First, this is a retrospective study. However, the selection bias may be negligible because only randomly assigned patients were included and there was marginal difference in the surgical techniques owing to the relatively short study period from November 2010 to March 2012. Second, our sample size was limited by the size of the original PONV and IOP trials, and follow-up data were not available for 2 patients. Our analysis was thus restricted to 64 patients in the TIVA group and 62 patients in the VA group. Although it may seem that the sample size is limited, we consider this to be a sufficiently influential outcome, since this study was based on data from a period of 7-9 years over which patients were enrolled and randomly assigned. Third, the volatile agents in the two original trials were different (desflurane in the PONV trial and sevoflurane in the IOP trial). However, no differences were found between the two volatile anesthetics following subgroup analysis. Finally, anesthetic induction was also performed using a bolus injection of propofol in the VA group. However, the influence of small amounts of propofol during the induction period in the VA group is considered to be insignificant based on the primary outcomes of previous studies where intraoperative IOP and PONV were significantly better in the TIVA group.

## Conclusions

This study was the first report on the long-term follow-up of prostate cancer recurrence after RALP under TIVA with propofol/remifentanil and VA with sevoflurane or desflurane/remifentanil. Our findings demonstrate that both TIVA with propofol/remifentanil and VA with sevoflurane or desflurane/remifentanil have comparable effects on oncologic outcomes in patients undergoing RALP. Additionally, we believe that the type of cancer should be considered as a potential factor in the clinical decision of the choice of anesthetic for cancer surgery. However, for further validation of our findings, additional prospective trials should be undertaken that will closely monitor and control the influence of opioids on oncologic outcomes.

## Figures and Tables

**Table 1:** Demographics and perioperative variables.

Variables	VA group (n = 62)	TIVA group (n = 64)	P value
Age, years	62.9 (7.1)	63.9 (8.0)	0.497
Height, m^2^	168.1 (5.2)	167.9 (6.9)	0.776
Weight, kg	69.3 (10.3)	69.1 (9.4)	0.920
ASA physical status			0.702
I	26 (42%)	29 (45%)	
II	36 (58%)	35 (55%)	
Sevoflurane/Desflurane, n	31/31		
Anesthesia time, min	190.4 (40.4)	192.1 (45.2)	0.821
Pneumoperitoneum time, min	114.7 (36.3)	118.9 (40.8)	0.546
Intraoperative input & Output			
Total fluid, mL	1731 (532)	1669 (683)	0.573
Colloid, mL	146 (258)	107 (186)	0.399
Blood loss, mL	442 (281)	375 (220)	0.193
Urine output, mL	167 (121)	224 (177)	0.069

Values are presented as means (SD) or numbers of patients (%). VA, volatile anesthesia; TIVA, total intravenous anesthesia; ASA, American Society of Anesthesiologists.

**Table 2:** Incidence of recurrence and pathological variables.

Variables	VA group (n = 62)	TIVA group (n = 64)	*p*-value
BCR	28 (45.2%)	25 (39.1%)	0.481
Positive radiographic progression	5 (8.1%)	3 (4.7%)	0.488
Initial PSA level, ng/mL	12.8 (12.2)	14.2 (18.0)	0.606
Gleason score			0.474
6	18 (29.0%)	24 (37.5%)	
7	30 (48.4%)	30 (46.9%)	
>=8	14 (22.6%)	10 (15.6%)	
Surgical margin status			0.722
negative	30 (48.4%)	33 (51.6%)	
positive	32 (51.6%)	31 (48.4%)	
Pathological Tumor stage			0.575
2	30 (48.4%)	37 (57.8%)	
3a	27 (43.6%)	23 (35.9%)	
3b	5 (8.1%)	4 (6.3%)	
Lymph node metastasis			> 0.999
negative	60 (96.8%)	61 (95.3%)	
positive	2 (3.2%)	3 (4.7%)	

Values are presented as means (SD) or numbers of patients (%). VA, volatile anesthesia; TIVA, total intravenous anesthesia; BCR, biochemical recurrence; PSA, prostate specific antigen.

**Table 3:** Univariate and multivariate analysis of risk factors for BCR after RALP (n = 126).

Variables	Univariate		Multivariate	
	HR (95% CI)	P value	HR (95% CI)	P value
Group	0.940 (0.548 - 1.613)	0.823	1.227 (0.660 - 2.283)	0.518
Age, year	1.002 (0.968 - 1.038)	0.904		
Height, m^2^	0.969 (0.924 - 1.017)	0.204		
Weight, kg	1.005 (0.977 - 1.033)	0.741		
ASA physical status				
I	1			
II	1.171 (0.680 - 2.018)	0.570		
Anesthesia time, min	0.994 (0.988 - 1.001)	0.082		
Pneumoperitoneum time, min	0.993 (0.985 - 1.000)	0.053		
Intraoperative input & Output				
Fluid input, mL	1.000 (1.000 - 1.001)	0.677		
Colloid input, mL	1.002 (1.001 - 1.003)	0.003	1.002 (1.000 - 1.003)	0.011*
Urine output, mL	1.001 (1.000 - 1.002)	0.331		
Blood loss, mL	1.001 (1.000 - 1.002)	0.412		
Initial PSA level, ng/mL	1.028 (1.015 - 1.041)	<0.001	1.025 (1.007 - 1.044)	0.006*
Gleason score				
6	1			
7	3.120 (1.353 - 7.194)	0.008	1.053 (0.313 - 3.539)	0.934
>=8	8.182 (3.424 - 19.551)	<0.001	1.630 (0.444 - 5.982)	0.461
Surgical margin status				
Negative	1			
Positive	2.967 (1.648 - 5.341)	<0.001	1.806 (0.893 - 3.653)	0.100
Pathological Tumor stage				
2	1			
3a	4.079 (2.159 - 7.704)	<0.001	2.262 (0.855 - 5.987)	0.100
3b	8.388 (3.477 - 20.239)	<0.001	4.217 (1.207 - 14.735)	0.024*
Lymph node metastasis				
Negative	1			
Positive	3.073 (1.100 - 8.587)	0.032	0.607 (0.154 - 2.395)	0.476

BCR, biochemical recurrence; RALP, robot-assisted radical prostatectomy; HR, hazard ratio; CI, confidence interval; VA, Volatile anesthesia; TIVA, total intravenous anesthesia; ASA, American Society of Anesthesiologists; PSA, prostate specific antigen.

## Abbreviations

RALP: robot-assisted laparoscopic radical prostatectomy
TIVA: total intravenous anesthesia
VA: volatile anesthetics
PONV: postoperative nausea and vomiting
IOP: intraocular pressure
BCR: biochemical recurrence
CO_2_: carbon dioxide
MAP: mean arterial pressure
BIS: bispectral index score
PSA: prostate specific antigen
HRs: hazard ratios
CIs: confidence intervals
HIF: hypoxia-inducible factor
MOR: µ-opioid receptors
